# Dissemination of periodic mammography and patterns of use, by birth cohort, in Catalonia (Spain)

**DOI:** 10.1186/1471-2407-8-336

**Published:** 2008-11-17

**Authors:** Montserrat Rue, Misericordia Carles, Ester Vilaprinyo, Montserrat Martinez-Alonso, Josep-Alfons Espinas, Roger Pla, Pilar Brugulat

**Affiliations:** 1 Biomedical Research Institut of Lleida (IRBLLEIDA), Lleida, Catalonia Spain; 2 University of Lleida, Lleida, Catalonia Spain; 3 Rovira i Virgili University, Reus, Catalonia Spain; 4 Bellvitge Institute for Biomedical Research (IDIBELL), Hospitalet de Llobregat, Catalonia Spain; 5 Catalan Cancer Plan. Department of Health, Catalonia Spain; 6 Catalan Institute of Health, Terres de l'Ebre Region, Catalonia Spain; 7 Catalan Health Department, Catalonia Spain

## Abstract

**Background:**

In Catalonia (Spain) breast cancer mortality has declined since the beginning of the 1990s. The dissemination of early detection by mammography and the introduction of adjuvant treatments are among the possible causes of this decrease, and both were almost coincident in time. Thus, understanding how these procedures were incorporated into use in the general population and in women diagnosed with breast cancer is very important for assessing their contribution to the reduction in breast cancer mortality. In this work we have modeled the dissemination of periodic mammography and described repeat mammography behavior in Catalonia from 1975 to 2006.

**Methods:**

Cross-sectional data from three Catalan Health Surveys for the calendar years 1994, 2002 and 2006 was used. The dissemination of mammography by birth cohort was modeled using a mixed effects model and repeat mammography behavior was described by age and survey year.

**Results:**

For women born from 1938 to 1952, mammography clearly had a period effect, meaning that they started to have periodic mammograms at the same calendar years but at different ages. The age at which approximately 50% of the women were receiving periodic mammograms went from 57.8 years of age for women born in 1938–1942 to 37.3 years of age for women born in 1963–1967. Women in all age groups experienced an increase in periodic mammography use over time, although women in the 50–69 age group have experienced the highest increase. Currently, the target population of the Catalan Breast Cancer Screening Program, 50–69 years of age, is the group that self-reports the highest utilization of periodic mammograms, followed by the 40–49 age group. A higher proportion of women of all age groups have annual mammograms rather than biennial or irregular ones.

**Conclusion:**

Mammography in Catalonia became more widely implemented during the 1990s. We estimated when cohorts initiated periodic mammograms and how frequently women are receiving them. These two pieces of information will be entered into a cost-effectiveness model of early detection in Catalonia.

## Background

In the European Union, age standardized breast cancer mortality declined by 7% from 1988 to 1996 [1] and by 1.7% from 1997 to 2002 [2,3]. In Catalonia (Spain), breast cancer was the leading cause of cancer death in women during 1997–1998. After a sustained increase starting in 1975–1976, mortality has declined 2.0% annually since 1989–1990 [4]. The dissemination of early detection by mammography and the introduction of adjuvant treatments are among the possible causes of this decrease, and both were almost coincident in time. Thus, understanding how these procedures were incorporated into use in the general population and in women diagnosed with breast cancer is very important for determining which part of breast cancer mortality reduction was attributable to early detection and which part to improved breast cancer treatments.

In the United States of America (USA), several groups modeled the effectiveness of mammography screening between 1975 and 2000 [5]. One of the common inputs of the models was the screening history of the cohorts alive during the studied period, which was analyzed by Cronin *et al *[6-8] at the National Cancer Institute (NCI). Cronin *et al *used survey and mammography registry data to develop a comprehensive model of mammography dissemination and use in the USA. Cronin's model consists of two parts describing screening dissemination and usage in US cohorts between 1975–2000. The first part describes the initial screening distribution, while the second models repeat screening behaviors. Together they tell us how many and how often women are being screened by age and birth cohort.

At the present time, there is interest in assessing the impact and cost-effectiveness of breast cancer early detection programs in Catalonia (Spain). The goal of this article is to model the dissemination of periodic and repeat mammography behavior in Catalonia from 1975 to 2006. We have been inspired by Cronin *et al*'s work although we have used a different methodological approach.

## Methods

In Spain there is a National Health System (NHS), financed mainly by taxes, which provides universal and free health coverage, including early detection of breast cancer. The NHS is organized on a regional basis and each Autonomous Region has the responsibility for organizing and providing health services to their population within the general principles of the NHS. Catalonia is an autonomous region of Spain which has approximately one sixth of the Spanish population. By the year 2007, the Catalan Health Service was providing services to 7 million inhabitants, including 3.5 million women. The Catalan Breast Cancer Screening Program (BCSP) started gradually at the beginning of the 1990s providing biennial mammography screening tests, with the target population being women 50–64 years old. Since the year 2000, women older than 64 are kept in the program until the age of 69. In 2007, in Catalonia, there were some 773 000 and 529 000 women in the 50–69 and 40–49 age groups, respectively. Before the start of and in addition to the BCSP, there has also been a certain degree of opportunistic breast cancer screening done in the public and private health care sectors. In the period 2001–2003, participation in the BCSP was 58% and the percentage of women having periodic mammograms was 72%. Therefore, around 14% of women in the BCSP target group received mammograms outside of the program. [9].

To study dissemination and patterns of use of periodic mammography, it would be desirable to have longitudinal registry data for different cohorts of women in the period of interest. This type of data does not exist in the majority of countries. Instead, cross-sectional population surveys may be used. In the USA, from the mid-1990s onward, there exists a longitudinal population registry available through the Breast Cancer Surveillance Consortium (BCSC) which Cronin *et al *used to model repeat screening behaviors. In Catalonia, there is no registry that collects population-based longitudinal data on mammography usage. Therefore, cross-sectional population surveys are also a source of data used to estimate repeat screening behavior.

We used cross-sectional data from three Catalan Health Surveys in the calendar years 1994 [10], 2002 [11] and 2006 [12]. The three Catalan Health Surveys were household interview surveys with a stratified multi-stage sampling design. Municipalities were selected with different probabilities based on their size within each of the eight Catalan Health Regions (strata). Then individuals were selected randomly from the municipal censuses. To minimize nonresponse when the selected sampling units were not found or declined to participate, they were replaced by extra units in the same age group, sex group, and neighborhood. The sample sizes of the Catalan Health Surveys were 15 000 individuals in 1994, 8 400 in 2002 and 18 126 in 2006.

We studied, first, how mammography has been disseminated in Catalonia by age and birth cohort, and second, patterns of repeated screening.

### Dissemination of periodic mammography, by age and birth cohort

**Figure 1:**
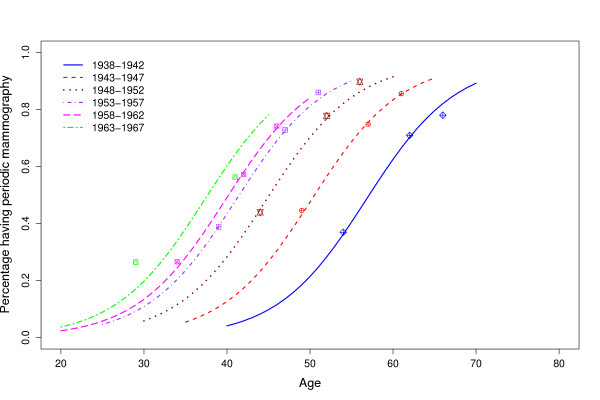
**Percentage of women that receive periodic mammograms by age and cohort of birth. Catalonia (Spain)**. The marks indicate the observed percentages at the Catalan health surveys in 1994, 2002, and 2006. The lines are the estimated percentages obtained using the mixed effects model $p=\frac{\phi_{1}}{1+exp[(\phi_{2}-age)/\phi_{3}]}$ with parameters *ϕ*_1 _and *ϕ*_3 _fixed and parameter *ϕ*_2 _random.

Table 1 shows the number of women interviewed, and the percentage of women using mammography periodically in Catalonia, by year of birth and age group. These percentages were obtained by applying the sampling weights to the responses reported in the survey. We used the commands *svy *from the statistical package *Stata *[13] to account for the sampling design. Percentages in Table 1 correspond to the cross-sectional observed values of the dissemination curves (see marks in Figure 1).

**Table 1:** Number of women interviewed and percent reporting having periodical mammograms

Birth cohort	Periodically screened								
	1994			2002			2006		
	age	n	%	age	n	%	age	n	%
1913–1917	77–81	268	3.35	-	-	-	-	-	-
1918–1922	72–76	358	5.63	-	-	-	-	-	-
1923–1927	67–71	459	10.67	75–79	155	18.27	-	-	-
1928–1932	62–66	471	20.94	70–74	209	37.56	74–78	431	33.14
1933–1937	57–61	521	24.97	65–69	251	51.53	69–73	504	49.26
1938–1942	52–56	403	36.93	60–64	188	70.90	64–68	398	77.98
1943–1947	47–51	510	44.59	55–59	264	74.82	59–63	482	85.55
1948–1952	42–46	532	43.92	50–54	290	77.77	54–58	542	89.72
1953–1957	37–41	517	38.78	45–49	293	72.79	49–53	566	85.96
1958–1962	32–36	539	26.61	40–44	313	57.17	44–48	636	74.15
1963–1967	27–31	460	26.51	35–39	309	25.15	39–43	691	56.25
1968–1972	22–26	527	14.05	30–34	242	17.69	34–38	684	25.23
1973–1977	17–21	588	6.20	25–29	326	14.86	29–33	764	17.64
1978–1982	-	-	-	20–24	353	5.56	24–28	721	11.12

Catalan Health Surveys, calendar years 1994, 2002, and 2006.

A mixed effects model was used to model the dissemination of periodic mammography, by age and birth cohort [14]. Since the observed data from the Health Surveys showed that the percentage *p *of women that use mammograms periodically increased with *age*, except in the older cohorts, the following logistic model was used:

$$p=\frac{\phi_{1}}{1+exp[(\phi_{2}-age)/\phi_{3}]}$$

where *ϕ*_1 _(*asym*) indicates the horizontal asymptote as *age *increases, *ϕ*_2 _(*xmid*) indicates the *age *value at which the response is *ϕ*_1_*/*2. It is the inflection point of the logistic curve. The scale parameter *ϕ*_3 _(*scal*) can be interpreted as the distance on the *x*-axis between the inflection point and the point where the dependent variable *p *is approximately 0.75 *ϕ*_1_. In other words, *ϕ*_1 _represents the proportion at which the dissemination curve levels off, or the highest proportion of women that receive periodic mammography, *ϕ*_2 _indicates the age at which half of the population that will end up receiving periodic screening is already receiving it, and *ϕ*_3 _indicates the difference in years between the age at which 3/4 of the population is receiving periodic mammograms and the age *ϕ*_2_.

Mixed effects models are suitable for analyzing grouped data and make it possible to incorporate both *fixed effects*, which are parameters associated with the entire population and *random effects *which are associated with individual units (in our analysis the individual units are *cohorts of birth*). A mixed effects model allowed us to break down the parameters *ϕ*_*i*_, *i *= 1, 2, 3 as *f*_*i *_+ *u*_*i *_where *f*_*i *_is the fixed effect and *u*_*i *_is the random effect, a value from a normally distributed variable with mean 0 and variance $\sigma_{u}^{2}$. The fixed effects part of the model summarizes the relationship between the variables for all cohorts together, whereas when the random effects are added to the fixed effects, the relationship between variables within each specific cohort is summarized. A small variance $\sigma_{u}^{2}$ for a specific random effect, indicates that the random effect is not significant and therefore there is no need to include it in the model. The interpretation is that all cohorts have similar behaviour with respect to this coefficient. In mixed effects models some of the coefficients may be treated as fixed and others as random, depending on the statistical significance of the random effects.

We used the package *nlme *(non linear mixed effects models) of the *R *software package for modeling [15]. Restricted maximum likelihood (REML) was used for parameter estimation. The significance of the fixed effects was tested using the *t*-test based on the REML estimate of the variance and the significance of random effects was tested using the likelihood ratio test.

### Repeat mammography behavior

The dissemination curves do not provide information on screening patterns, only on the fraction of women in the population that have periodic mammograms. We have used cross-sectional data from the Catalan Health Surveys [10-12] to estimate repeat mammography behavior. The proportion of women that reported having annual, biennial or longer intervals between mammograms were obtained, by age groups, from the 1994, 2002, and 2006 health surveys, taking into account the survey design. Intervals between mammograms longer than two years are named irregular intervals in this article.

## Results

### Percentage of women that receive mammography periodically, by age and birth cohort

Table 1 presents cross-sectional estimates of the percentage of women that received periodic mammograms by cohort, calendar year and age group. Cohorts born between 1938 and 1957 showed a period effect in the 1994 survey. They initiated periodic mammograms during the same calendar year but at different ages. Approximately 40% or more of these cohorts reported having periodic mammograms in 1994. In fact, in 1994, 37% of women in the 52–56 age group, 45% of the 47–51 group and 44% of the 42–46 group were exposed to periodic mammograms. By 2002 (as reported in the 2002 survey), these percentages had jumped to 70% or more, with the 1948–1952 cohort having the highest mammogram use. This cohort also reported the highest use of periodic mammograms (90%) in the 2006 health survey, at ages 54–58. The increase between 2002 and 2004 was markedly less than the increase between 1994 and 2002. It should be noted that the dramatic changes between 1994 and 2002 took place over eight years, twice the lenght of time as between 2002 and 2006. Eighty-five percent of women from both the 1943–1947 and the 1953–1957 cohorts indicated that they received periodic mammograms in 2006. Cohorts born between 1928 and 1937 experienced a decrease in the proportion of women that reported having periodic mammograms from the 2002 to the 2006 health surveys. There was also a slight decrease in the prevalence of periodic mammograms, for cohorts born in the 1963–1967 period, from 1994 to 2002.

Table 2 presents the mixed effects model for cohorts born between 1938 and 1967. Cohorts born before 1938 did not follow a logistic model since at older ages there was a reduction in the percentage of women receiving periodic mammograms. For cohorts born after 1967 there was not enough data to estimate the parameters. Random effects for the parameters *ϕ*_1 _(*asym*) and *ϕ*_3 _(*scal*) were not statistically significant, indicating that all the studied cohorts had similar values (Table 2, random effects section). The parameter *asym *= 0.968 indicates that approximately 97% of the women in each of the studied cohorts will end up receiving periodic mammograms. The parameter *ϕ*_2 _(*xmid*) which indicates the age at which half of the population is being screened periodically, had a significant random effect, indicating that this age changed across the studied cohorts. Thus, for cohorts born in 1948–1952, approximately half of the population was receiving periodic mammograms by age 45, for cohorts born in 1943–1947 this happened at age 50, and for cohorts born in 1938–1942 this happened at age 57. For cohorts born from the early 1960s onward, more than half of the women were already receiving periodic mammograms before the age of 40 (Table 2, random effect for parameter *ϕ*_2_).

**Table 2:** Mixed effects logistic model* for estimating the percentage of women receiving periodic mammograms by age and cohort of birth

**Fixed effects**			
Parameter	Value	Standard error	p-value
*ϕ*_1_(*Asym*)	0.968	0.065	< 0.001
*ϕ*_2_(*xmid*)	45.004	3.091	< 0.001
*ϕ*_3_(*scal*)	5.384	0.640	< 0.001

**Random effects**			
Parameter	Standard deviation	Residual	

*ϕ*_1_(*Asym*)	NS		
*ϕ*_2_(*xmid*)	6.697	0.002	
*ϕ*_3_(*scal*)	NS		

**Random effect for parameter *ϕ*_2 _(*xmid*) by cohort of birth**			
Cohort of birth	Random effect **	Fixed plus random ***	

1938–1942	11.771	56.78	
1943–1947	5.193	50.20	
1948–1952	-0.244	44.76	
1953–1957	-3.883	41.12	
1958–1962	-5.160	39.84	
1963–1967	-7.677	37.33	

NS: Not significant.

* The model is:

$p=\frac{\phi_{1}}{1+exp[(\phi_{2}-age)/\phi_{3}]}$ were *p *indicates the percentage of women receiving periodic mammograms, *ϕ*_1 _(*asym*) is the horizontal asymptote as *age *increases or the percentage at which the curve levels off, *ϕ*_2 _(*xmid*) indicates the *age *value at which approximately half of the population is receiving periodic mammograms and *ϕ*_3 _(*scal*) indicates the difference in years between the age at which 3/4 of the population is receiving periodic mammograms and the age *ϕ*_2_.

(**) The values indicate the departure from the fixed effect (45.0 years of age) for each cohort of birth. (***): The values can be interpreted as the age at which approximately 50% of the women will be receiving periodic mammograms.

Figure 1 is the graphical representation of the logistic curves. Marks in Figure 1 represent the observed data, obtained from the responses to the surveys, and lines represent the estimated dissemination curves using the model described in Table 2 with fixed parameters *ϕ*_1 _and *ϕ*_3 _and random parameter *ϕ*_2_. For women born between 1938 and 1952, mammography clearly had a period effect, meaning that they started to have periodic mammograms in the same calendar years but at different ages. Curves for cohorts born after 1952 appear closer, indicating that the incorporation of women into periodic screening had a similar age pattern between cohorts. More than 40% of women born in 1953–1957 were already receiving periodic mammograms at age 40. This percentage increased to 50 and 60 percent for cohorts born in 1958–1962 and 1963–1967, respectively. The curve for cohorts born in 1963–1967 was estimated with data from women younger than 45 years of age and should be interpreted with caution.

**Figure 2:**
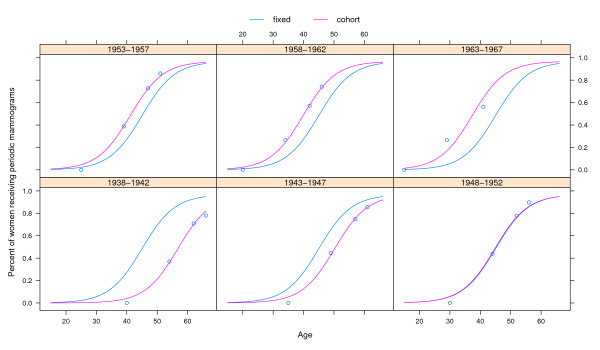
**Fixed and random effects obtained using a mixed effects model for the percentages of women that receive periodic mammograms, by age and cohort of birth. Catalonia (Spain)**. The fixed effects' curves represent the dissemination of mammography for all cohorts together, whereas the random effects' curves represent the dissemination of mammography for each specific cohort and are obtained using the corresponding cohort-specific *ϕ*_2 _parameter.

Figure 2 presents the estimated dissemination curves, by cohort of birth, distinguishing the fixed and random effects. The blue line is the curve obtained using the fixed effects and represents the modeling of all the cohorts together, whereas the pink lines are specific for each cohort (random effects) and are obtained using the corresponding cohort-specific parameter *ϕ*_2_. Location of cohorts below or above the overall dissemination curve indicates differences in the percentage of women that received periodic mammograms by age.

### Repeat mammography behavior

**Figure 3:**
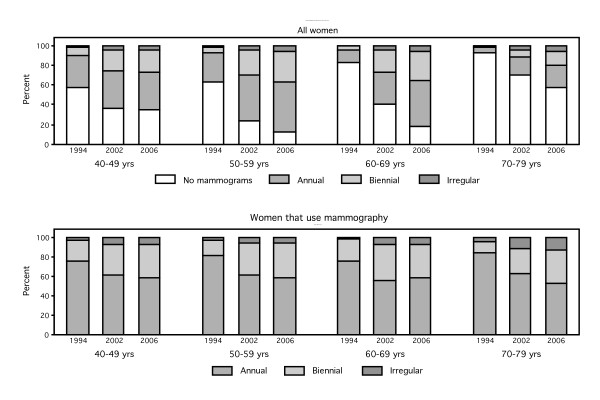
**a – Proportion of women that fall in the categories of not having periodic mammograms or having mammograms annually, biennially or irregularly, by age and period. **3b – Proportion of women that fall in the categories of having mammograms annually, biennially or irregularly among those that declared to have periodic mammograms, by age and period.

Figure 3a shows the proportion of women classified as not having mammograms or being annual, biennial or irregular screeners, by age and period. Figure 3b shows the proportions, by age and period, among the screening users. Women in all age groups have experienced an increase in mammography use over time, although women in the 50–69 age group have experienced the highest increase.

Eighty eight percent of women 50–59 years old and 83% of women 60–69 years old reported having periodic mammograms in the 2006 health survey. Approximately 50% of women in these age groups had annual mammograms, whereas 1/3 of the women 50–69 years old had biennial mammograms (Figure 3b).

In the 40–49 age group, the percentage of women with periodic mammograms increased from 43% in 1994 to 64% in 2002 and 2006. In this age interval, in the 2002 and 2006 health surveys, 40% of women reported having annual mammograms, 20% biennial and 5% irregular mammograms.

In the 70–79 age group only 6% of women reported having periodic mammograms in 1994 whereas in 2006 this percentage was 42%. Also, in this age group most of the women that have mammograms have them annually.

In summary, we have evidence from the three Catalan Health Surveys of a substantial increase over time in the use of mammography, and a higher proportion of women that have annual mammograms than biennial or irregular mammograms.

## Discussion

This paper studied the dissemination of periodic mammography and repeat mammography behavior in Catalonia (Spain), from 1975 to 2006. First, we modeled the percentage of women receiving periodic mammograms by age and cohort of birth. Second, we described patterns of repeated use over time. Third, we obtained one of the inputs that will be used in the near future for modeling the effectiveness of early detection and adjuvant treatment on the reduction of breast cancer mortality in Catalonia, Spain.

### Limitations

One of the limitations of our study is the fact that our data comes from self-reported information from a representative population sample. Recall bias may affect the information that individuals report in the interview. Self-reported information has commonly been used to study mammography utilization across populations and time periods. Bancej *et al *[16] used longitudinal panel data from a representative cohort of women to examine the prevalence of inconsistent self-reports of mammography utilization in Canada, as assessed in 1994–95 (baseline) and 1996–97 (follow-up). Among women who reported having a mammogram at baseline, 5.9% reported at follow-up that they had never had one. Among women reporting never using mamography at baseline and at least one mamography at follow-up, 17.4% reported their most recent mammogram as occurring prior to 1994–95 (baseline) and such responses were more common among women aged 70+ years and those in poorer health. Rivera *et al *[17] assessed the reliability of the self-reported lifetime number of mammograms, most recent mammogram date, and predictors of reliability in women veterans in the US. Reliability of the lifetime number of mammograms was 61% for exact consistency and 80% for consistency within one mammogram. Only 35% of women in the Rivera study were exactly consistent in reporting mammogram date and 55.6% were consistent within 3 months. White race/ethnicity, having a Bachelor's degree, reporting a healthcare provider's recommendation for a mammogram and having a screening mammogram were associated with consistency in reporting date. Although these studies point to potential measurement error, the fact that our study is derived from questions about utilization at the time of the survey, recall bias may have a lower impact in our data.

To support the validity of our data, on one hand we have other studies done in Catalonia that are consistent with our results. The BCSP has reported the percentages of women in the screening target group (aged 50 to 69) that either participated in the BCSP or have reported to the program that they had received recent mammograms, for the period 2001–2003 [9]. These values were consistent with the reported data of the 2002 Catalan Health Survey. Another survey of about 1600 women in the city of Barcelona in 1992, found that 27% of women aged 30–39 and 38% of women aged 40–49 reported having periodic mammograms [18]. These results also agree with our estimated curves (see ages 30–39 for cohorts born in 1953–57 and 1958–62, and ages 40–49 for cohorts born in 1943–47 and 1948–52). In addition, Segura *et al *interviewed around 8800 women aged 50 to 64 years in Barcelona in 1991, prior to initiating a population-based breast cancer screening program (which is part of the BCSP) [19]. Around 60% of these women reported having had screening mammography in the last 4 years, and mammography use was higher among women who were younger. These results are compatible with those obtained with our model (see dissemination curve for cohorts born in 1938–42). On the other hand, data in other countries, such as the US, suggests a similar pattern of mammography use, principally for the younger cohorts [6]. In the US, Cronin *et al *estimated that the percentage of women who had ever been screened for cohorts born in 1938–1942 was higher than 95%. On the other hand, in the US, cohorts born in 1948 or later had more than 50% of women ever screened before age 35. Even though Cronin *et al *studied the *percentage ever screened *and we have studied the *percentage with periodic mammograms*, we think that in both places a high percentage of women are regular mammography users when they reach the age of 40.

A second limitation arises from the Catalan Health Surveys' interviewees not being asked if their mammograms were for diagnosis or screening. Therefore, we have probably overestimated the cumulative distribution for the time to the first periodic mammogram for screening purposes. Cronin *et al *[6] estimated that 2% of the mammograms recorded in the Breast Cancer Surveillance Consortium were performed within nine months of the previous exam and therefore could be done for diagnostic reasons. Consequently, we think that there is minimal impact from overestimating the mammograms for screening purposes in the parameter estimates of the dissemination curves.

A third limitation occurs from the unlikely assumption of population stationarity that we made when combining cross-sectional data to estimate dissemination of mammography. In Catalonia, after a baby boom during the seventies, there were minor changes in the demographic structure between 1980 to 1994. The size of the population remained around 6 million inhabitants and mortality and fertility experienced a decreasing trend that resulted in an ageing population. From 1995 to 2006 there has been a dramatic increase in economic immigration, principally young individuals coming from less developed countries, that has produced a jump from 6 to 7 million inhabitants and modified the demographic age, sex, and socioeconomic structure [20]. Until now there has been no available data analyzing the impact of immigrants on mammography use in Catalonia. Since immigrants are mostly young, their impact on mammography utilization would be more marked in younger age groups. Some authors in Spain have reported higher usage of screening mammography in younger women with higher socioeconomic or educational status and private health insurance [18,21]. In consequence, the impact of immigration in Catalonia may have produced a decrease in the frequency of periodic mammography utilization in the younger cohorts, consistent with the decline that we have observed in our data.

A fourth limitation is that we use growth curves to study the use of periodic mammography, a phenomenon that can show a decreasing pattern at older ages. In fact, we have seen a decrease in the proportion of women that report having periodic mammograms in the oldest age groups. This finding is consistent with the guidelines for screening in our region, where women 70 years and older are no longer invited for periodic mammograms, free of charge, by the public health system. A way to avoid this limitation would be to restrict the analysis to women under 70. In fact, the curves that we could estimate corresponded to cohorts that were under 70 at the last health survey, in 2006. For earlier cohorts, we have presented the observed prevalences of periodic mammography use by age.

Fifth, our work was inspired by the work that Cronin *et al *did in the US, which adapted a standard diffusion of innovation model proposed by Rogers [6,22]. In the US, Cronin *et al *used data from seven national health interviews and estimated the parameters of the dissemination curve for each cohort separately. In Catalonia, there were only three national health surveys that provided data on mammography use. We started using the diffusion of innovation model and compared the results with a mixed effects model. Dissemination curves were similar with both methods, but since our mixed effects model combined data from all cohorts, it gave us more stable parameter estimates and also provided an interesting interpretation of how cohorts had differential patterns of use of periodic mammography over time.

### Dissemination of mammography

For the first part of the analysis, the mammography dissemination model in Catalonia, we used mixed effects models which incorporate both *fixed effects *and *random effects*. In most cases, random effects are associated with individual units drawn at random from a population. But mixed models can also be applied to data that present some type of correlation, like longitudinal or nested data, or in meta-analysis to assess heterogeneity of studies. Quoting Pinheiro and Bates: "*mixed models provide a flexible and powerful tool for the analysis of grouped data*" [14]. Since birth cohorts are affected by events that make individuals within cohorts more similar than individuals from different cohorts, it can be assumed that cohorts introduce a grouping characteristic in our data. Our mixed model estimated the relation between the percentage of women that use periodic mammography by age, assuming that this relation could vary among cohorts of birth. There are other examples in the literature where the cohort effect has been treated as a random effect. O'Brien *et al *used a mixed model to estimate age, period and cohort effects [23]. They treated the cohort effects as random effects and age and period effects as fixed. Assuming that the effects of membership in particular cohorts are variable instead of fixed, they solved the problem of identifiability of age-period-cohort models and, in addition, they showed how cohort characteristics can be used to explain the variance that is associated with cohorts and is independent of age and period.

We have based our analysis on the three available cross-sectional studies: the Catalan Health Surveys performed in 1994, 2002, and 2006. This fact limited the ability to estimate the parameters of the dissemination curves for the oldest and youngest cohorts that we present in Table 1. We have limited this analysis to cohorts born between 1938 and 1967 because the estimated parameters for these subgroups of cohorts were stable and precise.

Cohorts born during the 1940s and the beginning of the 1950s started to have periodic mammograms during the 1990s, at different ages. That indicates a period effect associated with the introduction of a new diagnostic test, which was also described by Cronin *et al *in the USA [6]. Dissemination curves for Catalan cohorts born after 1952 appear closer in the graph, indicating the end of the period effect and the incorporation of women into periodic screening at similar ages.

The Catalan BCSP covered the target population (women 50–64 years old) by the end of the 1990s and was extended to women 69 years old in the year 2000. In 2004, 61.2% of the invited women participated in the BCSP and 75.7% either participated in the BCSP or reported to the BCSP that they had received recent mammograms (non-published BCSP data). These values are slightly higher than the corresponding values reported by the BCSP for the period 2001–2003 [9]. The 2006 Catalan Health Survey shows an increase in the percentage of women that reported having periodic mammograms in the target group, being almost 90% in the 50–59 group and more than 80% in the 60–69 group.

There has been a dramatic increase in the use of periodic mammography at ages 40–49, an age interval not covered by the BCSP, for women born during the 1950s. This increase has stabilized near 75% for women born at the beginning of the 1960s, as Table 1 and Figure 1 show. Masuet *et al *[24] reported that Catalan women in the 40–49 age group had the highest prevalence of mammography use in 1994, but were surpassed by women in the 50–69 age groups in 2002. In addition, there has been an increase in mammography use over time, in women in their late 30s or early 40s, which can be measured by the change in the age at which approximately half of the cohort is already receiving periodic mammograms. This age went from 57 years for cohorts born in 1938–1942 to 37 years for cohorts born in 1963–1967. In cohorts born at the end of the 1950s, more than half of the women were receiving periodic mammograms at the age of 40. Therefore, opportunistic screening is being done in Catalonia, in young women, at ages where screening mammography is still controversial.

Finally, women in their early 30s, while in 1994 one in four reported periodic mammography use, in 2002 and 2006 this figure was one in six. Reasons for this decrease may have been, a) a change in the sociodemographic composition of these age groups due to the arrival of immigrant women, probably less exposed to preventive or screening practices, b) a higher level of consensus, for this age group, among health professionals regarding evidence and criteria for screening mammography, and c) the active role of the Catalan health authorities for adherence to the European guidelines [25]. In Catalonia, before the advent of the BCSP, private health services actively encouraged the use of mammography in young women.

### Repeat mammography behavior

Since there are no longitudinal registries of mammography in Catalonia, we have limited our analysis to a description of mammography usage patterns (non-user, annual, biennial, irregular) based on health surveys from 1994, 2002, and 2006.

Whereas the Catalan BCSP currently invites women in the 50–69 age interval for biennial mammograms, our results show that annual mammography is the most prevalent pattern in Catalan women of all age groups. According to our data, 50% of the Catalan women in the 50–69 age group receive annual mammograms and only one third receive biennial mammograms (Figure 2b), as recommended in the BCSP. Since about 2/3 of the invited women participate in the Catalan BCSP, there might be a group of women that receive mammograms both inside and outside the BCSP. If we assume that all the women 50–69 that receive biennial mammograms receive them in the BCSP, there may be a subset of women that participate in the BCSP irregularly and another fraction that receive additional mammograms in the public health system outside the BCSP or in the private health system.

It seems that in Catalonia periodic screening outside the BCSP is associated with higher socioeconomic status. And, from our data, it is easy to conclude that the majority of women that receive regular mammograms outside the BCSP receive annual mammograms. Bare *et al *[26] studied the behavior of women with respect to early detection of breast cancer prior to being invited to participate in a screening programme and factors associated with participation, in an area of Catalonia in the mid 1990s. More than 70% of the women reported that they had undergone mamography before the program was started. Around 60% of the women that reported having had mammograms received them less than 2 years previously. Factors associated with non-participation in the screening program were higher level of education, higher occupational skills or working at home, self- or gynaecological examination of breasts, and having received hormone replacement therapy. Borras *et al *[21] and Borrell *et al *[27] found that Catalan women who reported having voluntary private health insurance (one quarter of the women's population) in addition to the universal insurance that all resident women have, were more likely to have had a regular mammography.

## Conclusion

Mammography in Catalonia started during the 1980s but spread during the 1990s. The modeling of mammography dissemination using a mixed effects model has facilitated an understanding of when cohorts started using mammography on a regular basis. Currently, more than half of women are receiving periodic mammograms in Catalonia at age 40.

In Catalonia, the target population of the Catalan Breast Cancer Screening Program, 50–69 years of age, is the group that reports the highest use of periodic mammograms, followed by the 40–49 age group.

The majority of women that receive periodic mammograms have them annually.

Finally, we have estimated when cohorts started the use of periodic mammograms and how frequently women are receiving periodic mammograms. These two pieces of information will be entered into a cost-effectiveness model of early detection in Catalonia.

## Competing interests

The authors declare that they have no competing interests.

## Authors' contributions

MR: Co-developed the project that includes this study, performed statistical analysis, wrote drafts and obtained author feedback.

MC: Co-developed the project that includes this study, participated in writing and revising the manuscript.

EV: Provided statistical analysis of results and interpretation. Participated in the writing and revising of the manuscript.

MM-A: Provided statistical analysis of results and interpretation. Participated in the writing of the manuscript.

J-AE: Co-developed the project that includes this study. Participated in the writing of the manuscript and the interpretation of data.

RP: Co-developed the project that includes this study, participated in the interpretation of data.

PB: Participated in the interpretation of data from the Catalan Health Surveys. Revised the manuscript and provided feedback.

All authors read and approved the final version of the manuscript.

## Pre-publication history

The pre-publication history for this paper can be accessed here:


